# One Molecule, Many Faces: Repositioning Cardiovascular Agents for Advanced Wound Healing

**DOI:** 10.3390/molecules29122938

**Published:** 2024-06-20

**Authors:** Anna Gościniak, Anna Stasiłowicz-Krzemień, Bożena Michniak-Kohn, Piotr Fiedor, Judyta Cielecka-Piontek

**Affiliations:** 1 Department of Pharmacognosy and Biomaterials, Poznan University of Medical Sciences, Rokietnicka 3 Str., 60-806 Poznań, Poland; agosciniak@ump.edu.pl (A.G.); astasilowicz@ump.edu.pl (A.S.-K.); 2 Department of Pharmaceutics, Ernest Mario School of Pharmacy, Rutgers-The State University of New Jersey, Piscataway, NJ 08854, USA; michniak@pharmacy.rutgers.edu; 3 Center for Dermal Research, Rutgers-The State University of New Jersey, Piscataway, NJ 08854, USA; 4 Department of General and Transplantation Surgery, Medical University of Warsaw, 02-008 Warsaw, Poland; piotr.fiedor@wum.edu.pl

**Keywords:** wound healing, repositioning, cardiovascular agents, topical formulations

## Abstract

Chronic wound treatments pose a challenge for healthcare worldwide, particularly for the people in developed countries. Chronic wounds significantly impair quality of life, especially among the elderly. Current research is devoted to novel approaches to wound care by repositioning cardiovascular agents for topical wound treatment. The emerging field of medicinal products’ repurposing, which involves redirecting existing pharmaceuticals to new therapeutic uses, is a promising strategy. Recent studies suggest that medicinal products such as sartans, beta-blockers, and statins have unexplored potential, exhibiting multifaceted pharmacological properties that extend beyond their primary indications. The purpose of this review is to analyze the current state of knowledge on the repositioning of cardiovascular agents’ use and their molecular mechanisms in the context of wound healing.

## 1. Introduction

Around 2% of individuals in developed countries grapple with chronic wounds at some point in their lives [1]. In Europe, as per data from the European Wound Management Association (EWMA), there is a reported prevalence of 3–4 chronic wounds per 1000 individuals, coupled with an annual incidence of 4 million patients [2,3]. Shifting the focus to the United States, chronic wounds impact 10.5 million U.S. Medicare beneficiaries, signifying an increase of 2.3 million since the 2014 update [4]. Notably, these wounds exert a tangible effect on the quality of life for nearly 2.5% of the entire U.S. population, with a more pronounced impact on the elderly demographic. Examining the varied realm of wounds reveals a range marked by distinct characteristics, each presenting specific challenges in the healing process. Acute wounds, typically resulting from trauma or accidents, demand immediate attention due to their sudden onset and potential complications. Chronic wounds, such as diabetic ulcers and pressure sores, arise from underlying health conditions, posing prolonged healing challenges and requiring specialized care [4]. Surgical wounds, intentionally created during medical procedures, present a distinct category, with their healing trajectory influenced by factors such as surgical technique, infection prevention, and postoperative care [5,6].

Civilization diseases, including diabetes, cardiovascular diseases, and obesity, significantly complicate the wound-healing process [7]. Individuals with diabetes, for instance, often experience impaired blood circulation and compromised immune function, rendering them more susceptible to chronic wounds and delayed healing [8]. Similarly, obesity can contribute to increased pressure on the lower extremities, predisposing individuals to the development of pressure ulcers [9]. Heart disease and its associated risk factors, such as dyslipidemia and hypertension, might be linked to impaired wound healing due to their effects on vascular health and inflammation [10]. Hypercholesterolemia, characterized by high levels of cholesterol in the blood, has been identified as a factor that can influence wound healing processes. Studies have shown that hypercholesterolemia can have detrimental effects on wound healing and angiogenesis, leading to delayed healing and impaired vascularization [11,12]. Hypercholesterolemia has been associated with increased hydroxyproline levels, worsened anastomotic wound healing, and impaired endothelial proliferation and angiogenesis, which are crucial processes in wound repair [11,13]. Hypertension has been identified as a factor that can impact wound healing processes. Studies have shown that hypertension is associated with changes resembling aberrant cutaneous wound healing phases that characterize pathological scar development [14]. Additionally, hypertension has been linked to delayed wound healing in specific patient populations, such as hypertensive patients undergoing total hip arthroplasty [15]. The effect of hypertension on wound healing outcomes has been observed in clinical settings, where non-responsive wounds were more likely to be hypertensive compared to responsive wounds [16]. Furthermore, the effects of hypertension on abdominal wall healing have been studied, with untreated hypertension surprisingly not impairing indicators of wound healing in abdominal wounds [17]. In the context of chronic wounds, such as venous leg ulcers, hypertension plays a role in the healing process. The standard of care for venous ulcer treatment includes addressing venous hypertension through compression therapy and maintaining a moist wound-healing environment [18,19]. Comorbidities like arterial hypertension have also been suggested to influence wound healing outcomes in patients with chronic leg ulcers [20]. Lifestyle diseases not only contribute to the prevalence of wounds but also amplify the complexity of their management.

In recent years, the concept of medicinal-product repurposing has emerged as a dynamic and promising field within the realm of medical research [21,22]. This innovative approach involves redirecting existing pharmaceutical agents towards novel therapeutic applications, presenting a unique avenue for therapeutic discovery and development. One particularly intriguing area of investigation is the repurposing of agents traditionally employed in the treatment of cardiovascular diseases for localized wound healing. The medications commonly prescribed for cardiovascular and vascular disorders exhibit multifaceted pharmacological properties that extend beyond their primary indications. Recent studies suggest that these medicinal products may harbor an untapped potential in the management of local injuries, specifically in wound healing processes [23,24,25]. The understanding that the active substances within cardiovascular medications could positively influence tissue regeneration and wound closure has spurred a growing interest in exploring their applications beyond cardiovascular disorders. Exploring innovative approaches, such as repurposing cardiovascular agents for wound care, also holds promise in mitigating some economic challenges. Leveraging existing pharmaceuticals with proven safety profiles may offer a cost-effective alternative for managing wounds. These medicinal products, known for their anti-inflammatory or pro-angiogenic effects, could reduce the need for specialized dressings and frequent changes [26,27,28]. Repurposing cardiovascular agents not only enhances therapeutic efficacy but also has the potential to alleviate financial strains associated with traditional wound care.

**Figure 1:**
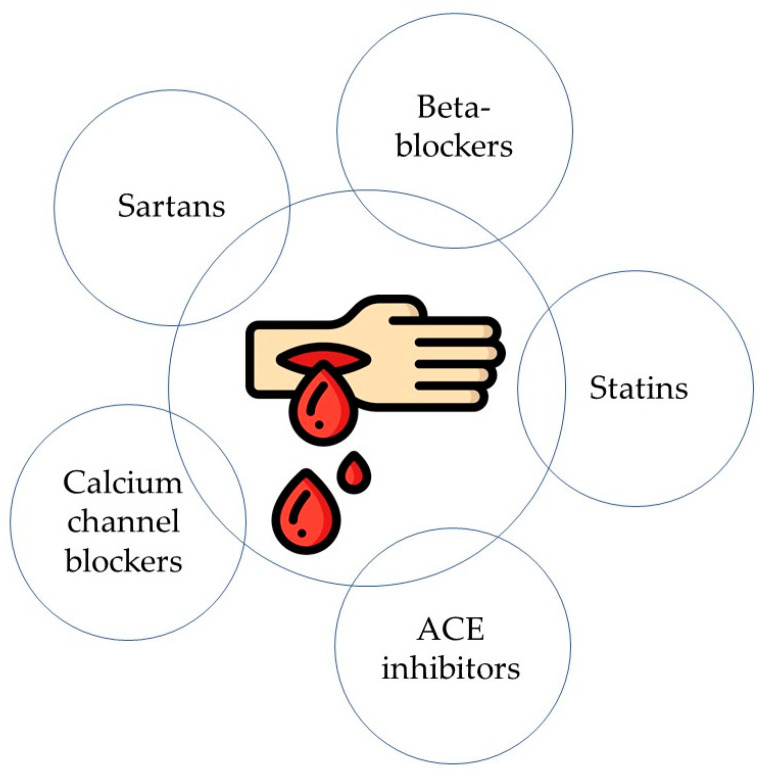
Therapeutic classes that can be repositioned for wound treatment.

This review aims to comprehensively examine the current state of knowledge on medicinal products repurposing, focusing on the use of cardiovascular agents in the context of wound healing. The groups discussed include those for which there are exhaustive reports on the impact of topical application on wound treatment. These groups include sartans (angiotensin II receptor blockers), statins, beta-blockers, calcium channel blockers, and angiotensin-converting enzyme (ACE) inhibitors (Figure 1). The molecular mechanisms underlying these effects on tissue repair are discussed, and the preclinical and clinical evidence that supports their potential in advancing the field of wound therapy is examined.

## 2. Applications of Sartans in the Treatment of Wounds

**Figure 2:**
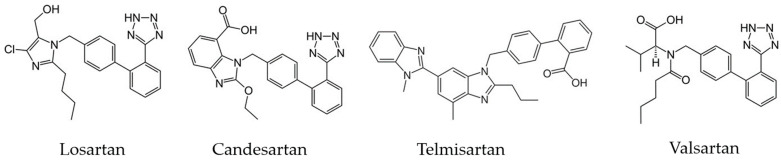
Chemical structures of sartans.

Sartans (Figure 2), also known as angiotensin II receptor blockers, are a class of medicinal products widely used for the treatment of hypertension, heart failure, and other cardiovascular disorders [29,30]. They block the action of angiotensin II, a hormone that constricts blood vessels, thereby causing them to dilate, and, hence, they help in lowering blood pressure. Sartans are used as the second choice drug, after angiotensin-converting enzyme (ACE) inhibitors, for the treatment of hypertension [30]. Moreover, they have neuroprotective potential that has been studied in vivo and in vitro [31,32]. The discovery of these compounds dates back to the 1970s and 1980s, when researchers sought ways to inhibit the renin–angiotensin–aldosterone system, which is crucial for regulating blood pressure and fluid balance in the body. The first sartan, losartan, was developed by scientists at DuPont Pharmaceuticals and approved by the FDA in 1995, marking a breakthrough in hypertension treatment by offering an alternative to ACE inhibitors, which often caused chronic cough [33]. With a favorable safety profile and fewer side effects, such as cough and angioedema, sartans have become essential in managing hypertension and cardiovascular diseases, with examples including losartan, valsartan, and irbesartan.

### 2.1. Effects of Sartans on Wound Healing

Kamber et al. [34] investigated the impact of losartan on tissue regeneration in diabetic mice with impaired wound healing. Using a streptozotocin-induced diabetic mouse model, losartan accelerated wound repair and normalized stromal responses, which exhibited a beneficial role in diabetic wounds. Losartan-treated diabetic mice showed improved wound healing, reduced inflammation, and normalized tissue remodeling without affecting blood glucose levels or body weight. Analyses of wound sections revealed accelerated tissue regeneration, reduced fibrosis, and improved extracellular matrix remodeling. The presence of myofibroblasts and increased vascularization in losartan-treated diabetic mice contributed to enhanced wound contraction.

Yahata et al. [35] revealed that angiotensin II in human keratinocytes and fibroblasts revealed significantly enhances cell migration. This effect was mediated through the activation of angiotensin II type 1 receptor (AT1R). Inhibition of AT1R using losartan attenuated the migratory response, underscoring the in vitro significance of this receptor. In the in vivo setting, AT1R knock-out mice were employed to elucidate angiotensin II’s contribution to wound healing. The absence of AT1R resulted in a pronounced delay in wound closure, highlighting the in vivo importance of angiotensin II. A further exploration revealed that angiotensin II facilitates keratinocyte and fibroblast migration in the wound bed, promoting efficient wound healing. Molecular investigations focused on the epidermal growth factor receptor (EGFR) signaling pathway. Angiotensin II triggered AT1R-dependent EGFR transactivation, a process that is crucial for both in vitro and in vivo cell migration. The shedding of heparin-binding epidermal growth factor (HB-EGF) emerged as a central mechanism driving angiotensin II-induced migration. The inhibition of AT1R with losartan, HB-EGF with CRM197, and EGFR with erlotinib effectively abrogated this migratory response in both in vitro and in vivo settings.

In a study performed by Abadir et al. [36], the topical administration of valsartan and losartan demonstrated efficacy in accelerating wound healing. Valsartan and losartan suppressed inflammatory cytokines, upregulated transforming growth factor beta 1 (TGF-β1), and promoted collagen deposition within the wound site. The application of 1% valsartan gel demonstrated significant benefits, leading to accelerated closure time and increased tensile strength in diabetic and aged mice, with validation in older diabetic pigs. Furthermore, wounds treated with 1% valsartan gel exhibited enhanced mitochondrial content, collagen deposition, and activation of various molecular markers related to wound healing.

### 2.2. Effect of Sartans on Scar Formation

The effect on scar formation is also an interesting issue from the point of view of drug repositioning. Fang et al. [37] investigated the potential antifibrotic effects of angiotensin-converting enzyme inhibitors on scar formation. The study involved culturing mouse NIH 3T3 fibroblasts with different angiotensin-converting enzyme inhibitor concentrations, revealing reduced fibroblast proliferation, suppressed collagen and transforming growth factor β1 (TGF-β1) expression, and downregulated phosphorylation of SMAD2/3 and growth factor beta-activated kinase 1 (TAK1) pathways in vitro. Furthermore, the study used a rat scar model, demonstrating that scars treated with ACEs, particularly ramipril or losartan, exhibited not only narrower dimensions than controls but also enhanced re-epithelialization and neovascularization, along with organized granulation tissue formation. Zhao et al. [38] studied a novel losartan cream fortified with chitosan and asiaticoside, comparing its efficacy against established treatments for scarring. Using a mouse scar model, losartan cream demonstrated superior anti-scarring effects, followed by the chitosan asiaticoside cream. Mechanistic investigations revealed inhibition of TGF-β1, collagen, and Smad expression, coupled with reduced Smad phosphorylation, both in vivo and in vitro.

Kurt et al. [39] investigated the effects of valsartan and enalapril, angiotensin-converting enzyme inhibitors (ACEIs), on pathological scar formation in a rabbit ear wound model. Enalapril (0.75 mg/kg/day) in the first group and valsartan (10 mg/kg/day) in the second group were administered for 40 days, alongside a control group (without treatment). Both medications significantly inhibited pathological scar formation, influencing fibroblast count, capillary count, collagen ratio, organization, and epithelial thickness. While the scar elevation index showed no significant difference between enalapril and control, valsartan demonstrated greater efficacy in reducing fibroblast count and epithelial thickness. Valsartan and enalapril emerged as effective inhibitors of pathological scar formation, suggesting their potential therapeutic roles in addressing scar-related histomorphological changes.

Huang et al. [23] explored the effectiveness of losartan, an angiotensin II receptor blocker, in inhibiting human hypertrophic scar fibroblasts (HSFs). Losartan demonstrated significant inhibition of both the proliferation and migration of HSFs, showing potential in preventing hypertrophic scar (HS) formation. Personalized microneedle patches of losartan prepared by stereolithographic 3D printing successfully penetrated porcine skin and rabbit hypertrophic scars, leaving visible channels for drug delivery. Losartan-loaded microneedles dissolved rapidly, facilitating efficient drug release into the skin. In treating rabbit hypertrophic scars, the personalized losartan-loaded microneedles significantly reduced scar hyperplasia, decreased the scar elevation index (SEI), and inhibited collagen deposition compared to other treatment groups.

In a pilot single-blind, placebo-controlled study, losartan potassium ointment (5%) demonstrated efficacy in alleviating keloid and hypertrophic scars [40]. Thirty adult participants, randomly assigned to losartan or placebo treatment groups, underwent a three-month treatment regimen, followed by a 6-month follow-up. VSS (Vancouver Scar Scale) scores significantly decreased in the losartan group for both keloid and hypertrophic scar patients. Moreover, losartan treatment led to a significant reduction in vascularity and pliability, suggesting its potential as a therapeutic intervention for scar management. An ongoing clinical trial [41], initiated on 7 June 2023 and registered under the identifier NCT05893108 on ClinicalTrials.gov, is currently investigating the efficacy of treating keloids. The trial is comparing the effects of 5% losartan potassium in ethosomal gel to 10% triamcinolone acetonide injections in 46 participants with keloids over a 12-week period, with assessments at baseline, 4 weeks, 8 weeks, and 12 weeks. This research aims to evaluate the potential anti-keloid effects of losartan potassium by targeting the angiotensin II receptor involved in inflammation, proliferation, and fibrosis.

### 2.3. Novel Formulation of Sartans in Wound Healing

In response to the need for innovative wound treatment solutions, researchers have explored advanced formulations incorporating liposomes, fibers, and nanoparticles to enhance the efficacy of wound care. Liposomes, lipid-based vesicles, are utilized to encapsulate therapeutic agents, facilitating controlled release and improved bioavailability of substances at the wound site [42]. These microscopic vesicles offer a protective environment for bioactive compounds, enhancing their stability and promoting targeted delivery. Nanoparticles, at the nanoscale level, exhibit unique properties that can be harnessed to enhance the delivery and absorption of therapeutic agents, contributing to the development of more effective and suitable wound care solutions [43].

In a study by Nidadavolu et al., valsartan amphiphiles were self-assembled into filamentous structures, acting as a scaffold for wound beds and enabling a steady release of valsartan over 24 days [44]. In Zucker Diabetic Fatty rats, two applications of this sustained-release valsartan filament hydrogel resulted in accelerated wound closure. By day 23, all val-filament treated wounds had completely closed, in contrast to one closure in the placebo group. Mechanistically, the treatment influenced proteins associated with cell adhesion and energetics pathways, downregulated TGF-β signaling pathway mediators, and increased mitochondrial metabolic pathway intermediates.

El-Salamouni et al. used valsartan solid lipid nanoparticles for treating uncontrolled diabetic foot ulcers [45]. The optimized formulation was incorporated into a hydroxyl propyl methyl cellulose (HPMC) gel for convenient administration. In vitro and in vivo characterizations demonstrated a small particle size, high entrapment efficiency, and sustained drug release. The obtained gel displayed notable antibacterial effects, reducing biofilm mass formation for Gram-positive and Gram-negative bacteria.

In vivo studies on diabetic rats revealed improved wound healing through various pathways, including cyclooxygenase-2 (COX-2), nuclear factor kappa B (NF-κB), nitric oxide (NO), transforming growth factor beta (TGF-β), matrix metalloproteinases (MMPs), and vascular endothelial growth factor (VEGF). A histological examination confirmed re-epithelization in treated ulcers, along with reduced collagen.

Ilomuanya et al. studied the potential of polylactic acid (PLA)-based electrospun nanofibers loaded with hyaluronic acid, valsartan, and ascorbic acid for chronic wound healing [46]. The presence of valsartan significantly influenced the re-epithelization rate in diabetic rats. Notably, the scaffolds reduced inflammatory cell infiltrates compared to controls. Conventional treatment and scaffolds with ascorbic acid demonstrated lower re-epithelization rates (59.45 ± 1.69% and 62.01 ± 1.68%, respectively) than those with HA-valsartan hydrogel (85.5 ± 1.7%).

El-Salamouni et al. [47] addressed burn wound treatment by developing liposomes with pentoxifylline–valsartan integrated into a gel matrix. The study aimed to co-deliver hydrophilic pentoxifylline and lipophilic valsartan through a nano-based liposomal system for topical use. The research systematically explored the impact of varying phospholipid amounts on co-entrapment efficiency, identifying the optimal composition. The resulting formulation demonstrated nanometric size, commendable entrapment efficiency, and sustained release, promising enhanced burn healing. The study also investigated the therapeutic potential of pentoxifylline–valsartan co-delivery on the high-mobility group box 1 (HMGB-1)/Toll-like receptor (TLR) pathway.

A summary of animal studies using sartans is shown in Table 1.

## 3. Applications of Beta-Blockers in the Treatment of Wounds

**Figure 3:**
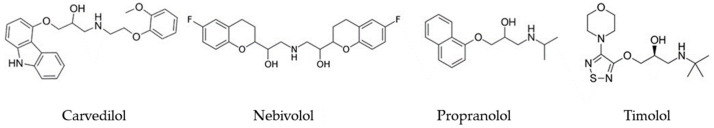
Chemical structures of beta-blockers.

Beta-blockers (Figure 3), also known as beta-adrenergic receptor blockers, discovered in the 1960s, are a class of medicinal products widely used in the management of various cardiovascular conditions such as coronary artery disease, heart failure, and arrhythmias [48,49,50]. They have been shown to reduce mortality and morbidity in patients with heart failure with reduced ejection fraction [48]. Additionally, beta-blockers have demonstrated efficacy in preventing migraine and tension-type headaches [51]. Studies have also indicated their potential benefits in reducing the risk of severe exacerbations in patients with chronic obstructive pulmonary disease [52]. Beta-blockers exert their effects through various mechanisms, including the blockade of adrenergic receptors and reduction of heart rate, which may contribute to their ability to reduce mortality in certain patient populations [53]. Beta-blockers have attracted interest for repurposing in wound healing, as studies have demonstrated their potential to modulate the inflammatory and proliferative phases of wound healing, delay wound contraction and re-epithelialization, and improve cutaneous wound healing in diabetic and chronically stressed animal models [54,55,56,57]. Furthermore, the widespread clinical use of beta-blockers for the treatment of hypertension and cardioprotection may alter catecholamine responsiveness, providing a potential avenue for their therapeutic application in wound healing [58]. Timolol is currently used in ophthalmology to lower intraocular pressure and control eye disorders such as glaucoma. Studies on the use of timolol for wound healing have shown promising results compared to other beta-blockers, suggesting its significant potential in this regard. Researchers have explored its effects on wound closure rates, inflammation control, and tissue regeneration. These findings highlight timolol as a promising option for improving the healing of wounds.

### 3.1. Effects of Beta-Blockers on Wound Healing

Ulger et al. [59] compared the effects of dexpanthenol and nebivolol on wound healing. Thirty rats were divided into three groups—a control group with no treatment, a second group treated with dexpanthenol cream, and a third group treated with 5% nebivolol cream. A 2 cm linear incision was made on the rats’ skin, and wound areas were measured on specific days. On the 21st day, all wounds were excised and histologically evaluated. Both dexpanthenol and nebivolol groups showed higher wound healing rates than the control group (*p* < 0.05), with no significant difference between the dexpanthenol and nebivolol groups, suggesting that nebivolol and dexpanthenol have comparable effects on wound healing.

Another study investigated the synergistic effects of nebivolol hydrochloride and chitosan on tissue regeneration [60]. Nebivolol hydrochloride-loaded chitosomes were prepared using the ionic gelation method, with chitosan lactate and sodium tripolyphosphate. Cell culture studies demonstrated significantly higher fibroblast proliferation compared to drug suspensions and the blank formula. An in vivo evaluation showed the superior efficacy of nebivolol hydrochloride-loaded chitosomes in wound healing, as evidenced by histopathological examination indicating enhanced wound proliferation and non-significant differences in collagen deposition after 15 days compared to intact skin.

The group of Zheng et al. [61] investigated the effectiveness of 1% propranolol cream applied intralesionally in diabetic wounds. Fifty-six spontaneously diabetic mice were divided into a propranolol-treated group and a control group. Wound sizes were consistently smaller in the propranolol group throughout the 21-day evaluation period. The propranolol group exhibited increased epidermal growth factor (EGF) protein expression and regulated angiogenesis, with lower vascular endothelial growth factor (VEGF) expression and increased NG2 proteoglycan. Matrix metallopeptidase (MMP)-9 expression initially favored the propranolol group but later shifted in the control group. In conclusion, intralesional administration of 1% propranolol cream demonstrated potential for promoting re-epithelialization and regulating angiogenesis in diabetic wounds. Freiha et al. studied the impact of propranolol, timolol, and minoxidil on full-thickness thermal skin burns in a Wistar rat model [62]. Fifty rats underwent specific interventions for 14 days: topical vehicle (control), topical silver sulfadiazine, oral propranolol with topical vehicle, topical timolol 1% cream, and topical minoxidil 5% cream. Timolol used topically effectively prevented necrosis, facilitated healing, and boosted antioxidant capacity.

In a case study, a 68-year-old patient with a chronic wound on the right sole, unresponsive to conventional treatments, experienced significant improvement and complete healing within four weeks following the application of a 1% propranolol-hydrochloride cream three times daily [63].

The oral administration of beta-blockers was also studied. Propranolol is used in severe infantile hemangiomas [64]. Propranolol was found to be safe and well-tolerated in neonates with infantile hemangioma, with observed adverse effects predominantly mild and manageable, and no serious adverse events were reported [65]. Other beta-blockers were also studied. Atenolol in treating ulcerated infantile hemangiomas shortened the average time to complete ulcer healing to 12.2 weeks from ulceration onset and 8.1 weeks from initiation of oral treatment, accompanied by improved hemangioma activity scores and generally mild side effects [66]. Moreira et al. administered atenolol preoperatively to 80 patients to maintain heart rate below 60 bpm and continued until the 15th postoperative day [67]. The results showed significantly lower average blood pressure and heart rate in the atenolol group, with no instances of expansive hematoma observed, in contrast to several cases in the control group. A case series of oral atenolol administered to 46 infants with infantile hemangiomas resulted in complete involution in 61.8% and partial regression in 38.2%, while maintaining a favorable safety profile with mild transient side effects observed in 23.9% of cases (primarily diarrhea and mild sleep disturbance) [68]. Not all studies on the oral administration of beta-blockers were positive. Beta1 and beta2 adrenoceptor blockades, through propranolol (50 mg/kg) and atenolol (100 mg/kg) administered orally for 14 days, delay cutaneous wound healing in male Wistar rats by affecting leukocyte migration, cell proliferation, myofibroblastic differentiation, and mast cell migration and reducing MMP levels [69]. In contrast, alpha1 and alpha2 adrenoceptor blockades, through phentolamine (20 mg/kg) administered orally for 14 days, did not significantly delay wound contraction or re-epithelialization. In a study published in 2023, the impact of orally administered propranolol (50 mg/kg) and metoprolol (50 mg/kg) over a period of 14 days on wound healing in 18 Sprague-Dawley rats using incision and excision wound models was studied [70]. Both drugs were found to decrease tensile strength, delay wound contraction and re-epithelialization, increase inflammatory infiltrate, and reduce collagen density and hydroxyproline levels, indicating that β-blockers delay wound healing through mechanisms involving β1-receptors.

### 3.2. Clinical Studies on Timolol

Much of the research conducted focuses on timolol, whose history of topical use has led researchers to expand this application. Many of these studies are clinical trials.

The multi-center study, conducted from 2016 to 2019, investigated the effectiveness of topical timolol (0.5%) in treating 55 chronic wounds across 39 patients with various etiologies [71]. Timolol drops were applied at one drop per cm^2^ of wound area, with variable frequencies. Patients stopped concurrent therapies, except for standard care. The results revealed 34 wounds that were completely healed, 15 that were improved, 4 that showed no change, and 2 that were worsened.

In another study, the impact of topical timolol on wound healing post-CO_2_ laser resurfacing was investigated [72]. In a 68-year-old subject, one forearm received timolol, while the other served as a control with standard care. The results showed quicker healing, reduced inflammation, and decreased erythema in the timolol-treated wounds by week 2, compared to the 4 weeks needed for the control. The study suggests that combining topical timolol with standard care accelerates re-epithelialization after laser resurfacing. However, caution is advised due to the potential systemic absorption of timolol.

Two cases of chronic, longstanding wounds were effectively treated with topical timolol 0.5% (Timoptic; Aton Pharma, Lawrenceville, NJ, USA), as an adjunct therapy [73]. The first case involved an elderly woman with persistent ankle ulcers despite various treatments, while the second case featured a woman with a non-healing foot ulcer. Both cases saw successful closure within 7-to-8 weeks of topical timolol application. Additionally, three other patients with chronic wounds exhibited improvement and, in three cases, achieved complete healing with topical timolol.

The Waldman et al. [74] article discusses the management of refractory hypergranulation, a common complication after dermatologic surgery, using timolol ophthalmic gel-forming solution. Despite routine wound care and topical silver nitrate, some cases remain challenging. The authors proposed twice-daily application of timolol maleate ophthalmic gel-forming solution 0.5% for up to 14 days. They present two cases where hypergranulation, unresponsive to standard care, and resolved after treatment with topical timolol.

The case report prepared by Tang et al. [75] described favorable results obtained from the application of topical timolol, i.e., 0.5% drops on the refractory wound. The 43-year-old patient, who had been dealing with the chronic wound on her left mid-back for 15 months, experienced rapid epithelialization and healing over the course of 8 weeks. Despite failing to respond to routine wound care and topical recombinant human platelet-derived growth factor, the wound saw complete epithelialization after the patient applied three or four drops of topical timolol daily. The wound exhibited rapid epithelialization and healing during this period. Timolol was well-tolerated, and no significant adverse events were reported.

In two cases of junctional epidermolysis bullosa in 1-year-old children with chronic wounds in the nail bed and neck fold, applying timolol maleate 0.5% eye drops twice daily under occlusion proved highly effective [76]. Despite prior use of potent topical steroids and silicone dressings, significant healing (100% and 80%) was achieved after 3 and 8 weeks, respectively. Timolol, a β2-adrenergic receptor antagonist, showed promise in promoting wound healing, especially in areas where impaired keratinocyte migration is common in junctional epidermolysis bullosa patients.

The study performed by Valluru et al. [77] investigated the use of topical timolol 0.5% in treating chronic non-healing foot ulcers. The research involved 100 participants. The inclusion criteria encompassed age over 18, non-healing ulcers persisting for more than 6 weeks, and bacteriologically sterile wounds. The topical application of timolol demonstrated a reduction in ulcer size on days 15 and 30.

In Thomas et al.’s [78] study, the use of topical timolol promoting the healing of chronic leg ulcers, specifically focusing on chronic venous and diabetic ulcers, was investigated. The study included 60 patients, with 30 in the study group treated with topical 0.5% timolol maleate solution, along with antibiotics and dressings, and 30 in the control group receiving only antibiotics and dressings. The mean percentage change in ulcer area at 4, 8, and 12 weeks was substantially higher in the group treated with topical timolol (25.29%, 43.77%, and 61.79%) compared to the control group (11.92%, 22.40%, and 29.62%).

One case report involved a 40-year-old male with a vasculitic ulcer on his lower left leg that did not respond to conventional treatments [79]. The ulcer, present for 3 weeks, was characterized by a punched-out appearance, measuring 5 cm in diameter, with undermined borders and fibrinous exudate at the base. After unsuccessful attempts with prednisolone and dapsone, the patient showed remarkable improvement and complete healing following the application of topical timolol 0.5% ophthalmic drops, which were applied sparingly, five drops three times a day, directly onto the base and borders of the ulcer. Weekly evaluations demonstrated reduced inflammation and pain, and significant wound closure within six weeks. The treatment did not affect blood pressure or potassium levels.

In another study, a 92-year-old woman with chronic leg ulcers, unresponsive to various treatments, including ultrasonic wound debridement, was introduced to topical timolol solution based on previous successful cases [80]. After applying four drops of 0.5% timolol maleate ophthalmic solution daily for 6 weeks, the treated ulcer showed significant improvement, leading to the initiation of timolol treatment for the control ulcer. At the 12-week review, the initially treated ulcer had completely re-epithelialized. No adverse effects were reported.

A randomized controlled trial compared the effectiveness of topical timolol with saline in treating chronic venous leg ulcers and assessing the mean reduction in ulcer area after 4 weeks [81]. Twenty patients were randomly assigned to two groups, with group 1 receiving 0.5% topical timolol every alternate day for 4 weeks, and the control group receiving saline. Ulcer measurements were conducted weekly, and the healing rates were compared after 4 weeks. The results showed a mean reduction in ulcer size of 86.80% with timolol and 43.82% with saline after 4 weeks. Topical timolol demonstrated a substantial improvement compared to saline treatment.

In a prospective study performed by Michelangelo et al. [82] from January to June 2016, 82 patients with chronic refractory ulcers of various etiologies were treated with topical timolol 0.5%. The ulcers, present for at least 3 months, had not responded to standard care. Treatment involved applying timolol drops (one drop every 2 cm of wound area per day for 6 weeks) covered with polyurethane film and dressings. Significant improvement in area reduction (up to 98.75%) was noted in all groups except diabetic and pressure ulcers. Patient satisfaction was high, and adverse effects were mild to moderate.

In a phase-II randomized controlled trial, the effectiveness and safety of timolol maleate gel in the treatment of challenging-to-heal venous leg ulcers (VLUs) over a 12-week period was studied [83]. Forty-three patients with VLUs, present for ≥24 weeks and with ≥50% granulation tissue, were enrolled in the trial. Timolol-treated patients (n = 21) received sustained-release timolol gel (Timoptol^®^ LP 0.5%, Santen, Tampere, Finland) every 2 days, in addition to standard care, while the control group (n = 19) received standard care alone. The primary objective was to achieve a ≥40% reduction in ulcer area by week 12. The results indicated that 67% of timolol-treated patients met this endpoint, compared to 32% in the control group. No serious adverse events were reported. Using timolol maleate for challenging-to-heal venous leg ulcers turned out to be beneficial and safe, warranting confirmation through larger randomized phase-III studies.

### 3.3. Antibacterial Potential of Beta-Blockers

Beta-blockers have not yet been extensively studied for antibacterial activity, and there is limited work on this activity. Carvedilol displayed antibacterial activity against various bacteria, particularly *Staphylococcus aureus* and *Staphylococcus epidermidis* [84]. It induced changes in the fatty acid composition of *S. aureus* and increased the permeability of bacterial cell membranes, particularly in Gram-positive bacteria. The study by Zawadzka et al. [85] investigated the synergistic antibacterial activity of ciprofloxacin and carvedilol against *S. aureus*. Uma et al. designed and synthesized sulfonamide and carbamate derivatives of nebivolol, which exhibited promising antimicrobial and anti-inflammatory activities [67].

### 3.4. Novel Formulation of Beta-Blockers in Wound Healing

Nanofibers and microsponges are innovative materials with significant potential in wound healing applications [86]. Nanofibers, due to their large surface area and elasticity, can effectively absorb moisture from the wound, while maintaining a suitable environment for healing. They have been shown to reduce wound healing time and have the potential for treating different wound types, such as trauma, diabetic ulcers, and burns. Additionally, nanofibers can be loaded with wound-healing promoters to enhance their wound-healing ability [87]. On the other hand, microsponges, with their porous structure, can absorb excess secretions from the wound, promoting healing and reducing the risk of infection [88].

Zaeri et al. [89] explored the wound healing potential of propranolol incorporated into electrospun nanofibrous wound-dressing mats. Electrospun mats with 0, 2, or 4% propranolol were evaluated for physicochemical properties, propranolol release, and biocompatibility. The 4% propranolol mat was observed to have thin fibers, high porosity, good hydrophilicity, and minimal degradability. Propranolol release showed Fickian behavior. In vitro, 4% propranolol mats demonstrated excellent biocompatibility with 173% fibroblast viability on day 5. In vivo, these mats significantly reduced wound size, improved histopathologic characteristics, and lowered oxidative stress after 14 days.

The study by Pandit et al. [90] developed a nebivolol-loaded microsponge gel for targeted drug delivery to wounds, promoting moist wound management. Specific microsponge formulations showed 80% drug release in 8 h in vitro. Spherical, porous microsponges were incorporated into a Carbopol 934 (2%) gel. The gel characteristics were assessed, indicating a viscous nature (12,610 cP). In vivo studies on diabetic rats demonstrated significant wound closure by day 10, supported by histological findings. The microsponge gel, with prolonged drug release, provided accessibility to the wound area and an optimal moist wound management environment, showcasing effective wound healing in diabetic rats.

A summary of animal studies using beta-blockers is shown in Table 2.

## 4. Applications of Statins in the Treatment of Wounds

**Figure 4:**
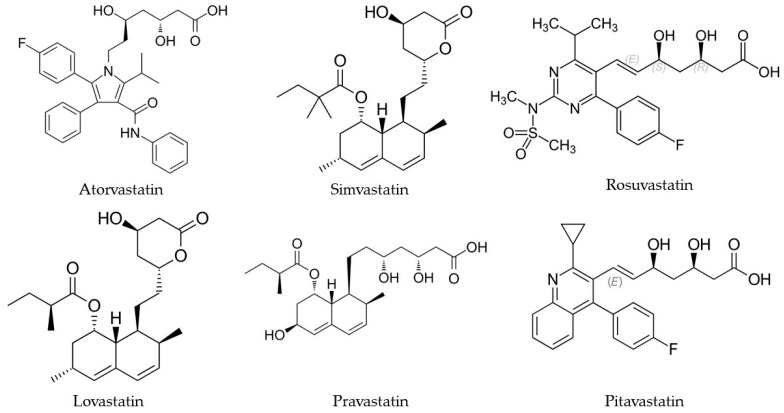
Chemical structures of the statins.

Statins (Figure 4), also known as HMG-CoA reductase inhibitors, are a class of medicinal products commonly used to treat heart disease. Their main therapeutic goal is to lower blood cholesterol levels by inhibiting the enzyme responsible for its production—HMG-CoA reductase. Statins, first discovered by Akira Endo in the 1970s, revolutionized cardiovascular disease prevention by inhibiting HMG-CoA reductase, a key enzyme in cholesterol synthesis. Following the approval of the first statin in 1987, subsequent statins demonstrated significant efficacy in lowering LDL cholesterol and reducing cardiovascular events. Despite occasional setbacks, such as the withdrawal of one statin due to safety concerns, statins have become a cornerstone in managing hypercholesterolemia [91]. Statin use has been a subject of extensive research globally, with studies focusing on various aspects, such as trends in utilization, associated risks and benefits, and factors influencing prescription patterns. Taken chronically in treatment regimens requires monitoring of renal function (eGFR and creatinine) [92]. Salami et al. [93] conducted a study on national trends in statin use in the US adult population from 2002 to 2013, revealing a significant increase in statin use among adults aged 40 and older. This increase was also observed in other studies, such as the research conducted by Mufarreh et al. [94], which indicated a rise in statin therapy for atherosclerotic cardiovascular disease from 2006 to 2016. These findings collectively demonstrate a global trend of escalating statin utilization over the stated years. Statins also have the prospect of repositioning themselves in the health market. The role of statins in healthcare goes beyond just controlling lipid levels. Statins also exhibit a number of other effects, such as anti-inflammatory, antioxidant effects, anti-cancer effects, and the ability to affect vascular endothelial function [95,96]. In addition to trends in utilization, some studies have also explored the association between statin use and specific health outcomes. Notably, statins exhibit potential benefits in conditions such as dermatitis, diabetes mellitus, nervous system diseases, coronary heart disease, inflammation, depression, and cancers [97,98,99,100]. Irvin et al. [101] conducted a systematic review and meta-analysis, revealing that statin use was associated with a reduced risk of ovarian cancer, particularly in populations with low pravastatin use. Furthermore, it was found that persistent and adherent statin use was associated with a decreased risk of Alzheimer’s disease [102].

At present, the sole route of statin administration approved by the Food and Drug Administration (FDA) is oral. Nevertheless, alternative administration methods have shown encouraging outcomes in diverse preclinical and clinical investigations. Studies have explored the topical application of statins for managing seborrhea, acne, rhinophyma, and rosacea, highlighting the expanding scope of their therapeutic implications beyond the traditionally approved oral route [103]. The wound healing activity of statins has been a subject of interest, with reports suggesting that statins may have wound healing activity by influencing molecular mechanisms of anti-inflammatory activity. Observations of improved wound healing ability in patients taking statins orally have been noted [104]. Studies have indicated that statins may be associated with diabetic foot ulcer healing and venous leg ulcer healing and may have an impact on fibroblast migration [105]. Furthermore, the effects of LDL cholesterol and pitavastatin treatment on fibroblast migration and wound healing have been explored, indicating potential effects on cell migration and wound healing in patients on statin therapy [106]. Reports have also suggested that statins play a role in fibrosis, which could affect the wound healing process following certain medical procedures [107]. These findings collectively suggest that statins may indeed have a role in promoting wound healing, potentially through various molecular and cellular mechanisms. However, further research is needed to fully understand the specific mechanisms and clinical implications of statins in wound healing.

The use of topical statins for wound treatment has gained attention due to their potential benefits in the healing process. Studies have shown that the topical application of statins, such as simvastatin and atorvastatin, can accelerate wound closure, promote angiogenesis, and enhance wound healing efficacy [24,108,109]. Additionally, the mechanisms underlying the beneficial effects of topical statins in wound healing have been elucidated, including the modulation of cellular processes involved in wound repair and the inhibition of pro-inflammatory factors [110]. Furthermore, research has indicated that statins possess antimicrobial activity against pathogens, which may help prevent wound infection and promote a favorable environment for healing.

### 4.1. Antibacterial Activity of Statins in Wound Healing

Effective wound healing involves managing bacterial colonization to prevent infections. Key bacteria, such as *S. aureus* (including MRSA) and *Pseudomonas aeruginosa*, pose significant risks [111]. The emergence of antibiotic-resistant strains, notably in *S. aureus*, underscores the urgency in controlling their growth. *Escherichia coli*, *Proteus mirabilis*, and *Acinetobacter baumannii/haemolyticus* are further strains of bacteria found in infected wounds. Data emphasize the predominance of Gram-negative bacteria in the studied population, with specific focus on these mentioned species [112]. The challenge extends to a mixed bacterial population, complicating wound care. In the era of antibiotic resistance, addressing wound infections requires strategic antimicrobial approaches. Topical agents and dressings with antiseptics are pivotal. Regular dressing changes and vigilant monitoring for signs of infection, including resistance-associated complications, are essential for optimizing wound healing outcomes.

Several animal studies and human observations have consistently indicated a correlation between statin treatment and a reduced susceptibility to bacterial infections, leading to improved overall outcomes [97,113]. A meta-analysis focused on the antimicrobial and anti-inflammatory effects of statins suggested a potential positive association between statin use and better outcomes in preventing and treating various infections, particularly in individuals receiving solid-organ transplants [114,115]. Statins may also exhibit direct antibacterial activity. The study of Abdelaziz et al. [116] demonstrated that combining rosuvastatin with levofloxacin resulted in a synergistic antibacterial effect against *S. aureus*, showcasing promising potential in combating antibiotic resistance. A study conducted by Thangamani et al. [117] highlights the broad-spectrum antibacterial effectiveness of simvastatin against both Gram-positive, including MRSA, and Gram-negative pathogens. Particularly noteworthy is its capacity to suppress MRSA toxin production, decrease bacterial load in skin infections, and demonstrate anti-biofilm activity, indicating the potential repurposing of simvastatin as a topical antibacterial treatment for skin infections.

Results obtained by Wang et al. [109] indicated that simvastatin exhibited bacteriostatic activity against *S. aureus* at sub-inhibitory concentrations up to 8 h post-exposure. However, further elevated concentrations beyond the minimal inhibitory concentration (MIC) did not increase the growth inhibitory effect. Intriguingly, simvastatin demonstrated a concentration-dependent ability to inhibit *S. aureus* biofilm formation. In an excisional mice wound model, the topical application of simvastatin at its MIC accelerated wound healing and facilitated bacterial clearance of *S. aureus*-contaminated wounds. Furthermore, in vitro studies have shown that statins, particularly simvastatin, have intrinsic antibacterial activity against oral and perioral microorganisms, including *Porphyromonas gingivalis*, a key pathogen in periodontal disease [118,119].

Hannachi et al. [120]’s study explored how statins impact the antibacterial effect of washed platelets against *S. aureus*. Statin-treated platelets exhibited a significant and dose-dependent potentiation of antibacterial activity. Flow cytometry revealed increased platelet activation with fluvastatin, reversed by the GPIIb/IIIa antagonist tirofiban. These findings suggest that statins enhance platelet-mediated defense against *S. aureus*, potentially contributing to their benefits in treating infective endocarditis. Further research is needed to elucidate molecular mechanisms and assess in vivo implications.

Only one clinical study focused on the topical antibacterial effect of statins. A randomized controlled trial conducted by Ahmadvand et al. [121] showed that topical simvastatin was associated with a greater decrease in acne severity as compared with those of oral and placebo groups.

### 4.2. Effects of Statins on Wound Healing

Statins, a class of medicinal products commonly used to lower cholesterol levels, have been found to affect various pathways involved in angiogenesis and lymphangiogenesis. Statins have been shown to modulate the expression of angiogenic factors, such as VEGF, and to exert pro-angiogenic effects through the activation of the mitogen-activated protein kinase/extracellular signal-regulated kinase (MAPK/ERK) and phosphatidylinositol-3-kinase/protein kinase B (PI3K/Akt) signaling pathways [122]. Furthermore, statins have been implicated in promoting endothelial cell proliferation and migration, which are essential processes in angiogenesis. However, the precise molecular mechanisms through which statins exert their effects on lymphangiogenesis require further investigation.

The study by Suzuki-Banhesse et al. [123] investigated the impact of atorvastatin on tissue repair following acute injury in healthy rats. Rats were categorized into four groups: placebo-treated, topical atorvastatin-treated, oral atorvastatin-treated, and topical plus oral atorvastatin-treated. Macroscopic examination revealed significant reductions in lesion areas for tested groups compared to the placebo group. A protein-expression analysis showed increased expression of cell-growth pathway-related proteins (insulin receptor substrate 1 (IRS-1), phosphoinositide 3-kinase (PI3K), protein kinase B (Akt), glycogen synthase kinase 3 (GSK-3), endothelial nitric oxide synthase (eNOS), vascular endothelial growth factor (VEGF), and extracellular signal-regulated kinase (ERK)), and anti-inflammatory cytokine interleukin-10 (IL-10) in atorvastatin-treated groups on various days. Conversely, pro-inflammatory cytokines interleukin-6 (IL-6) and tumor necrosis factor alpha (TNF-α) were downregulated. The findings suggest that atorvastatin accelerated tissue repair in rats and modulated expressions of proteins and cytokines associated with cell-growth pathways, highlighting its potential as a therapeutic option for promoting wound healing. In a study performed by Asai et al. [24] on genetically diabetic mice, the topical application of simvastatin was found to significantly accelerate the skin wound healing process. Simvastatin demonstrated a dual effect by enhancing both angiogenesis and lymphangiogenesis in the wound area, addressing crucial aspects of impaired wound healing in diabetes. Simvastatin promoted the infiltration of macrophages, which produced Vascular Endothelial Growth Factor C (VEGF-C), supporting angiogenesis and lymphangiogenesis. In vitro studies revealed that simvastatin directly influenced capillary morphogenesis and exhibited an antiapoptotic effect on lymphatic endothelial cells. Overall, the findings suggest that locally applied simvastatin holds therapeutic potential for improving wound healing in conditions with impaired microcirculation, such as diabetes. The research led by Sawaya et al. [110] explored the potential of mevastatin, a naturally occurring statin, as a novel treatment for diabetic foot ulcers. Using in vitro, human ex vivo wound models, and in vivo models, the study demonstrated that mevastatin accelerated wound closure by reversing the effects of farnesyl pyrophosphate and cortisol, inhibitors of healing. Notably, mevastatin inhibited cortisol synthesis in keratinocytes, modulating the glucocorticoid receptor pathway. The study highlights mevastatin’s ability to reverse c-Myc overexpression, a biomarker associated with non-healing wounds, and induce the long noncoding RNA Gas5, promoting epithelialization and angiogenesis. Overall, these findings suggest the potential repurposing of statin, specifically mevastatin, for topical treatment of diabetic foot ulcers, presenting a promising therapeutic option. Moreover, the authors reported that mevastatin promoted healing by targeting caveolin-1 to restore EGFR signaling, indicating the potential of statins to modulate critical signaling pathways involved in wound healing [124]. In a study by Ramhormozi et al. [125], the focus was on evaluating the wound healing potential of topical simvastatin and its influence on the protein kinase B/mammalian target of rapamycin (Akt/mTOR) signaling pathway in burn wounds. The experimental design included six groups of rats subjected to different treatments, revealing that topical simvastatin application significantly expedited wound healing, enhanced collagen deposition, and increased myofibroblast population. A gene expression analysis demonstrated heightened levels of CD31 (an endothelial marker), VEGF (vascular endothelial growth factor), Akt (protein kinase B), mTOR (mammalian target of rapamycin), and p70S6K (ribosomal protein S6 kinase) in simvastatin-treated wounds. These genes play crucial roles in angiogenesis, cell proliferation, and protein synthesis, emphasizing the multifaceted impact of simvastatin on wound healing at the molecular level. Intriguingly, the positive effects of simvastatin were counteracted by rapamycin, a mTOR inhibitor, underscoring the crucial role of the Akt/mTOR signaling pathway in simvastatin-induced wound healing. In a study conducted by Xiang et al. [126], a novel mesh-like electrospun fiber loaded with atorvastatin was designed and found to significantly enhance paracrine secretion by bone marrow-derived mesenchymal stem cells promoting wound healing in vivo. The fabricated electrospun membrane demonstrated successful atorvastatin loading; induced optimal endothelial cell migration and angiogenesis; and increased the expression of key growth factors, VEGF, and basic fibroblast growth factor (b-FGF) through focal adhesion kinase (FAK) and protein kinase B (Akt) signaling pathways. The in vivo application of atorvastatin-loaded mesh-like membranes resulted in accelerated wound healing, improved neovascularization, and enhanced collagen reconstruction, suggesting the potential efficacy of drug-loaded electrospun matrices for regenerative medicine and artificial skin development.

### 4.3. Novel Formulation of Statins in Wound Healing

In the search for effective wound treatments, the focus has been on the topical application of statins, but their limited lipophilicity has presented significant challenges. Recent studies present a variety of approaches to address this problem, introducing innovative drug delivery technologies. Simvastatin is a promising wound-healing agent, but it faces limitations in skin permeability due to its lipophilic nature. To overcome these challenges, a study by Mousavi-Simakani et al. [127] investigated the application of nanostructured lipid carriers as a novel topical drug delivery system. These carriers were designed to improve skin adhesion, maintain integrity, sustain the release of simvastatin, and hence provide an effective solution for pressure ulcer treatment. In vitro studies demonstrated sustained drug release, with 87% release of simvastatin over 48 h. In vivo assessments on a rat skin model revealed that a simvastatin-loaded gel significantly improved pressure ulcer healing, reduced inflammation, and promoted skin regeneration compared to both drug-free nanostructured lipid carriers and conventional simvastatin gels. This suggested that simvastatin-loaded nanostructured lipid carriers with a particle size of 100 nm hold promise as an effective topical drug delivery system for sustained pressure ulcer treatment.

Saghafi et al. [128] conducted a double-blind randomized clinical trial to evaluate atorvastatin-loaded emulgel and a nanoemulgel for surgical wound healing and postoperative pain reduction. The study randomized participants (laparotomy patients) to atorvastatin-loaded emulgel 1% atorvastatin-loaded nanoemulgel 1% or placebo emulgel for 14 days. Both atorvastatin formulations significantly reduced Redness, Edema, Ecchymosis, Discharge, and Approximation (REEDA) scores on Days 7 and 14, with atorvastatin nanoemulgel showing a pain reduction on the Visual Analogue Scale (VAS). The study suggested the effectiveness of both atorvastatin-loaded emulgel and nanoemulgel in accelerating laparotomy surgical wound healing and relieving concomitant pain without significant side effects.

A sustained-release drug delivery system was developed by Yasasvini et al. [129] that involved incorporating simvastatin–chitosan microparticles into polyvinyl alcohol (PVA) hydrogels to enhance wound healing efficiency. The microparticles, optimized for entrapment efficiency and morphology, exhibited a maximum entrapment efficiency of 82%, with a mild interaction between the drug and chitosan confirmed by the FTIR study, where intra-molecular hydrogen bonding and a slight shift of bands were demonstrated. Hydrogels containing a 2.5 mg equivalent dose of simvastatin-loaded chitosan microparticles showed a maximum cumulative percentage drug release of 92% over 7 days. In vivo wound healing studies on Wistar rats demonstrated a significant reduction in wound area with low-dose simvastatin–chitosan microparticle-containing hydrogels compared to untreated animals and those treated with 1% simvastatin ointment. Overall, the incorporation of simvastatin–chitosan microparticles in PVA hydrogels demonstrated significant and optimized wound healing efficiency.

Morsy et al. [108] investigated the wound healing potential of atorvastatin through the use of a various topically applied gel and emulgel formulations. The study rigorously assessed various atorvastatin formulations, considering physical characteristics, rheological behavior, in vitro drug release, and ex vivo drug permeation. The atorvastatin gel formulations exhibited favorable physical properties, with distinct drug release profiles across gel, emulgel, and nanoemulgel. Notably, atorvastatin’s skin permeation significantly increased (*p* < 0.05) when incorporated into nanoemulgel. In vivo studies on wound-induced rats demonstrated that atorvastatin nanoemulgel achieved the highest percentage of wound contraction. Histopathological assessments 21 days after treatment showed a significant improvement in skin morphology.

By formulating simvastatin cubosomes for topical delivery, the study performed by Ahmed et al. [130] introduced an innovative strategy to enhance simvastatin’s skin permeation, thereby optimizing the wound-healing effects of cubosomes. Cubosomes are distinct from solid nanoparticles and are liquid crystalline particles formed through self-assembly [131]. Comprising specific surfactants and an appropriate water ratio, these microstructured particles exhibit unique and significant properties. Incorporating the optimal formulation into a cubogel dosage form exhibiting pseudoplastic rheological behavior, further supported enhanced drug permeation through excised rat skin compared to free simvastatin hydrogel. The in vivo rat studies provided conclusive evidence of the promising potential of simvastatin cubosomes as an effective remedy for wound healing.

The novelty of study by Xiang et al. [126] lies in the design of a mesh-like electrospun fiber loaded with atorvastatin, which has not been previously explored. This innovative approach creates a unique microenvironment that enhances the paracrine secretion of bone marrow-derived mesenchymal stem cells, leading to accelerated wound healing. The combination of mesh-like topography and drug release from the electrospun membrane represents a novel strategy in regenerative medicine, demonstrating the potential for functional electrospun membranes as promising dressings for traumatic skin defects and artificial skin development.

The study by Janipour et al. [132] suggests that a peptide hydrogel (thixotropic) containing simvastatin, designed for diabetic wound healing, exhibited promising results in terms of accelerating skin wound recovery effectively and safely in diabetic mice. The Fmoc–diphenylalanine hydrogel with incorporated simvastatin demonstrated an entangled nanofibrous microstructure, shear-thinning characteristics, and sustained drug release over 7 days. In diabetic mice, a simvastatin-containing hydrogel showed superior outcomes in terms of wound contraction, tissue regeneration, reduced inflammation, and enhanced re-epithelization compared to the unloaded hydrogel controls. These findings suggest that the sustained-release hydrogel formulation has the potential for topical wound therapy in diabetic patients, addressing a significant global health concern related to diabetic wound healing.

In the context of periodontal disorders, a study by Madi et al. [114] explored the potential benefits of intra-oral topical application of simvastatin/chitosan gel on the palatal donor site following free gingival graft procedures, aiming to assess its impact on wound healing and discomfort reduction in individuals with periodontal conditions. This investigation aimed to evaluate the effectiveness of applying simvastatin/chitosan gel intra-orally after free gingival graft procedures, revealing a significant reduction in wound-healing scores at days 3 and 7 post-surgery, along with decreased discomfort in the simvastatin/chitosan gel group compared to other studied treatments—simvastatin suspension, chitosan gel, and petroleum gel. The findings propose the potential utility of this topical application as a novel therapeutic strategy for improved healing and pain reduction in the palatal donor site following free gingival graft (FGG) procedures in dentistry.

The objective of research conducted by Aly et al. [133] was to develop a hydrogel incorporating polymeric nanoparticles of simvastatin for the purpose of topical wound healing. The polymeric nanoparticles of simvastatin were created using the nanoprecipitation technique to enhance both drug solubility and skin permeation, with subsequent evaluation of their key characteristics. Following this step, the obtained nanoparticles were introduced into a hydrogel base; the final formulation exhibited good uniformity, and the pH was deemed acceptable and compatible with the skin. Furthermore, the viscosity and spreadability ensured ease of application. The hydrogel exhibited a high drug content, efficient release (81% within 24 h), and significant ex vivo permeation (69.19% after 24 h). Application of the gel on rat wounds led to accelerated healing, as observed through complete epithelialization and minimal inflammatory cell infiltration by day 11. The findings suggest that the hydrogel loaded with SIM PoNPs holds promise for effective wound healing.

The study by Varshosaz et al. [134] aimed to create a hydroxypropyl methyl cellulose (HPMC)/chitosan gel containing polymeric micelles loaded with simvastatin and evaluate its effectiveness in rat wound healing. The optimized formulation exhibited a biphasic drug release with sustained profile over 96 h. The gel with nanomicelles demonstrated improved sustained drug release compared to nanomicelles alone. In an excision wound model on rats, the simvastatin-loaded micelles-gel group showed superior wound closure. A summary of animal studies using sartans is shown in Table 3.

**Table 3:** Animal studies that tested the efficacy of statins in wound healing.

Drug	Model	Method of Administration/ Dosage/Duration of Treatment	Findings	Author
Atorvastatin	Wistar-Hannover rat model of wound	Topical, topical + Oral mg/g atorvastatin/5% atorvastatin cream 14 days	Accelerated tissue repair in rats and modulated expressions of proteins and cytokines associated with cell-growth pathways.	Suzuki-Banhesse et al. [123]
Simvastatin	Diabetic C57BLKS/J-m+/+Leprdb mice model of wound	Topical 0.5% simvastatin in petroleum jelly 10 days	Significantly accelerated skin wound healing in genetically diabetic mice and enhanced angiogenesis and lymphangiogenesis.	Asai et al. [24]
Mevastatin	Pathogen-free pigs (Ken-O-Kaw Farms, Windsor, IL) model of wounds	Topical 250 μM mevastatin for 20 min after wounding 4 days	Accelerated wound closure, promoted epithelialization and angiogenesis, and modulated critical signaling pathways involved in wound healing.	Sawaya et al. [110]
Simvastatin	Wistar rats weighing 200–250 g model of burns	Topical 0.5% simvastatin in petroleum jelly, daily 14 days	Expedited wound healing, enhanced collagen deposition, increased myofibroblast population, and upregulated angiogenic genes; Akt/mTOR signaling pathway involvement.	Ramhormozi et al. [125]
Atorvastatin	Sprague-Dawley rat (180–220 g) model of pressure sores	Topical Mesh-like electrospun fiber loaded with atorvastatin (2.5, 25, and 250 mg) 14 days	Enhanced paracrine secretion by bone marrow-derived mesenchymal stem cells, accelerated wound healing, improved neovascularization, and enhanced collagen reconstruction.	Xiang et al. [126]
Simvastatin	Wistar rat model of wound	Topical 0.1% simvastatin-loaded NLCs gel, twice daily 14 days	Improved pressure ulcer healing, reduced inflammation, and promoted skin regeneration.	Mousavi-Simakani et al. [127]
Simvastatin	Wistar rat model of wound	Topical Polyvinyl alcohol hydrogels with 2.5 mg, 5 mg and 10 mg of simvastatin, once in 7 days 21 days	Enhanced wound healing efficiency, caused significant reduction in wound area, and improved neovascularization.	Yasasvini et al. [129]
Atorvastatin	Wistar rat model of wound	Topical 2.5% atorvastatin gel, emulgel, nanoemulgel, daily 21 days	Favorable physical properties, with distinct drug release profiles across gel, emulgel, and nanoemulgel. Notably, atorvastatin’s skin permeation significantly increased (*p* < 0.05) when incorporated into nanoemulgel.	Morsy et al. [108]
Atorvastatin	Wistar rat model of wound	Topical Simvastatin-loaded cubosomal gel, daily 14 days	Enhanced simvastatin skin permeation and optimized wound-healing effects.	Ahmed et al. [130]
Simvastatin	Diabetic mice	Topical (Peptide hydrogel)	Promising results in accelerating skin wound recovery in diabetic mice.	Janipour et al. [132]
Simvastatin	Wistar rat model of wound	Topical Polymeric nanoparticles in hydrogel (20 mg of simvastatin) 11 days	Accelerated wound healing with complete epithelialization and minimal inflammatory cell infiltration.	Aly et al. [133]
Simvastatin	Wistar rat model of wound	Topical Gel incorporated nanomicelles loaded with simvastatin (0.5%) change for a new one every 3 days 15 days	Sustained drug release and improved wound closure in excision wound model in rats.	Varshosaz et al. [134]

## 5. Other Cardiovascular Drugs

There have been attempts to explore the potential of cardiovascular drugs from other classes as well. However, these studies are relatively fewer in number, and their outcomes have generally been less effective, or based on non-human research models. Table 4 summarizes the in vivo studies of calcium channel blockers and ACEI on wound healing.

Calcium channel blockers were discovered in the mid-1960s, during experimental work on molecules being screened as coronary dilators, leading to the identification of the mechanism of calcium entry blockade by these drugs [135]. These compounds exert their effects by inhibiting voltage-dependent L-type calcium channels in various tissues, including vascular smooth muscle, cardiac muscle, and endocrine tissue [136]. These medications are widely used in the management of cardiovascular conditions such as hypertension, angina, and arrhythmias [137]. Calcium channel blockers are categorized into dihydropyridines and non-dihydropyridines. Previous studies have shown potential for both types of calcium channel blockers in promoting wound healing based on various animal models and in vitro research. Nifedipine and verapamil inhibit collagen and proteoglycan synthesis in vitro in uniaxially oriented mammalian fibroblast-populated collagen matrices [138]. Nifedipine also demonstrated potential in restoring epidermal barrier function by reversing disruption caused by sodium caprate in normal human epidermal keratinocytes, potentially involving correction of claudin-1 irregularities in tight junctions [139]. Nifedipine (2 mg/kg p.o.) and amlodipine (1 mg/kg p.o.) enhanced wound healing in normal and steroid-depressed conditions in rats, as indicated by the increased tensile strength of granulation tissue, possibly through improved collagen cross-linking and antioxidant activity [140]. Rats treated with 5% verapamil gel every 24 h for 15 days exhibited faster wound-closure rates, increased fibroblast density, higher volume densities of collagen bundles and vessels, and larger vessel diameters compared to the control [141]. Amlodipine 1% and a combination of amlodipine 1% with phenytoin 1% in rabbits with excisional cutaneous wounds accelerated wound closure compared to controls, with healing completed by the 13th and 9th days, respectively [142]. Azelnidipine, administered orally at 3 mg/kg/day, accelerated diabetic wound healing in rats by enhancing nitric oxide synthesis and improving histologic parameters after two weeks of treatment [143]. Topical nifedipine 3% ointment significantly improved the inflammatory phase in both normal and diabetic rats, while also enhancing the maturation phase, specifically in diabetic rats [144]. However, it did not significantly affect the proliferation phase in either group. Al-Dabbagh et al. studied 1% and 2% topical nifedipine ointments’ impact on TGF-β levels and wound healing in rabbits [145]. Nifedipine 1% significantly increased TGF-β levels on days 7 and 14, promoting faster wound closure compared to other groups. Conversely, nifedipine 2% showed no such effect. Chitosan-based polymeric powders containing nifedipine used on rats showed improved wound healing, with a higher tensile strength and faster epithelialization compared to controls [146]. Topical nifedipine on skin wound healing was also studied in pigs, focusing on polymorphonuclear cells, vascular proliferation, and collagen formation [147]. Eight wounds were created on each of the three pigs, treated with varying concentrations of nifedipine cream (1%, 10%, and 20%) or saline solution. Polymorphonuclear cell activity and vascular proliferation increased in nifedipine-treated wounds compared to controls. Collagen formation, however, decreased notably in one animal. In a randomized, double-blind, placebo-controlled clinical trial, 200 critically ill patients with stage I or II pressure ulcers were treated with either topical nifedipine 3% ointment or placebo twice daily for 14 days [148]. The study demonstrated that nifedipine significantly accelerated healing compared to placebo, as evidenced by a greater reduction in ulcer stage and surface area by days 7 and 14.

ACEIs were discovered in the 1970s as a result of research aimed at understanding the renin–angiotensin–aldosterone system and its role in regulating blood pressure [149]. These compounds work by inhibiting the conversion of angiotensin I to angiotensin II, a potent vasoconstrictor, leading to vasodilation and decreased blood pressure [150]. ACEIs have become a cornerstone in the management of hypertension, heart failure, and other cardiovascular conditions [151]. Studies highlight mixed outcomes of captopril’s effects on wound healing across different models and conditions. An in vitro study on human keratinocytes performed by Baroni et al. revealed that captopril significantly upregulated acetylcholinesterase expression, which enhances acetylcholine degradation and reduces its secretion [152]. These results suggest a potential mechanism linking ACEI to acantholysis, possibly mediated by altered acetylcholine levels in keratinocytes. Treating fibroblasts with captopril led to a significant decrease in prolidase activity accompanied by a parallel reduction in collagen biosynthesis, which may result from the suppression of prolidase activity through the inhibition of IGF-IR and alpha2beta1 integrin signaling [153]. In the animal model, a study evaluated captopril’s effects on partial thickness burn wound healing in rats [154]. The study found no positive impact on inflammation or wound healing. Local captopril treatment even reduced wound closure compared to systemic treatment and controls. In patients, the inhibition of collagen synthesis and deposition, also delaying wound healing by ACEI [155,156], in patients was noted. Some results might indicate that ACEI inhibitors may not promote wound healing, but their activity might have other uses. For example, ACEI have proven effective in the treatment of patients suffering from keloids [157,158].

Topical captopril showed positive results on wound healing in a study on New Zealand white rabbits, where full-thickness dermal wounds were treated with 5% captopril for seven days [159]. It also effectively prevented hypertrophic scar formation, likely by inhibiting collagen synthesis and modulating wound-healing processes [159]. Another study on rats investigated the effects of oral captopril (25 mg/kg/day and 50 mg/kg/day administered for 11 days) on wound healing, nitric oxide, and vascular endothelial growth factor levels in diabetic rats. Captopril-treated groups had higher nitric oxide levels and better wound healing scores compared to the diabetic control group. Vascular endothelial growth factor levels were also significantly higher in captopril-treated groups on day 5. The authors suggest that captopril may improve wound healing in diabetic rats. Zhao et al. studied another ACEI, perindopril, which was administered locally at the fracture site in ovariectomized rats with tibial fractures; it significantly enhanced bone healing by increasing bone formation and biomechanical strength, as well as improving the microstructural parameters of the callus [160].

**Table 4:** In vivo studies that tested the efficacy of other cardiovascular drugs.

Drug	Model	Method of Administration/ Dosage/Duration of Treatment	Findings	Author
Nifedipine and amlodipine	Healthy Wistar rats	Oral 2 mg/kg (nifedipine) 1 mg/kg (amlodipine) 9 days	Enhanced wound healing, increased tensile strength in granulation tissue.	Bhaskar et al. [140]
Verapamil	Male Wistar rats aged Between 2 and 3 months	Topical 5% gel 15 days	Accelerated wound closure rates, higher fibroblast density, increased collagen bundle and vessel volume densities, and larger vessel diameters.	Ashkani-Esfahani et al. [141]
Amlodipine and amlodipine with phenytoin	New Zealand rabbits	Topical 1% amlodipine/amlodipine 1% with phenytoin 1% 13/9 days	Accelerated wound closure (21 days non-treated, 13 days—amlodipine group, 9 days amlodipine/phenytoin group).	Hemmati et al. [142]
Azelnidipine	Male Sprague-Dawley diabetic rats	Oral 3 mg/kg 14 days	Faster wound closure, increased nitric oxide synthesis, and improved histologic parameters.	Bagheri et al. [143]
Nifedipine	Normal and diabetic male Wister rats	Topical 3% ointment 7 days	improved the inflammatory phase in both groups, enhanced the maturation phase in diabetic rats, did not significantly affect the proliferation phase in either group.	Cheraghali et al. [144]
Nifedipine	Healthy, mature male rabbits	Topical 1%, 2% ointment 7/14 days	1% ointment significantly increased TGF-β levels on days 7 and 14, faster wound closure; 2% ointment did not increase TGF-β levels, no effect on wound closure.	Al-Dabbagh et al. [145]
Nifedipine	Female Wister rats	Topical Chitosan-based polymeric powders 12 days	Improved wound healing, higher tensile strength, and faster epithelialization.	Samy et al. [146]
Nifedipine	Three healthy Pietrain pigs	Topical Nifedipine cream (1%, 10%, and 20%)	Increased polymorphonuclear cell activity and vascular proliferation. Collagen formation decreased in one animal.	Brasileiro et al. [147]
Captopril	Male Wistar rats	Systemic and topical 5% captopril solution 42 days	No positive impact on inflammation and in wound healing. Local treatment reduced wound closure compared to systemic treatment and controls.	Akershoek et al. [154]
Lisinopril	Case study, 71-year-old male patient with a venous leg ulcer	Oral lisinopril treatment	Delayed wound healing.	Buscemi et al. [155]
Captopril	Case report, 18-year-old female patient	Topical 5% solution in cold cream, Twice daily for 6 weeks	Marked improvement in keloid: reduced lesion height, eliminated redness and scaling, and reduced itchiness. No cutaneous or systemic side effects observed.	Ardekani et al. [157]
Enalapril	Case study, 40 patients with multiple keloids w	Intralesional injection 0.125 mg/mL, 30 units/session 3 sessions monthly 3 months	Significant improvement in keloid appearance.	Iannello et al. [158]
Captopril	New Zealand white rabbits	Topical 5% captopril 7 days	Increase in collagen organization, an 8.50% decrease in collagen organization scale was derived by captopril compared to the vehicle (70% ethanol and 30% propylene glycol).	Ardekani et al. [159]
Captopril	Male Sprague-Dawley diabetic rats	Oral 25 mg/kg/day and 50 mg/kg/day 11 days	Higher nitric oxide levels. Improved wound healing scores. Elevated vascular endothelial growth factor levels.	Zandifar et al. [161]
Perindopril	Female Sprague-Dawley rats (ovariectomized with tibial fractures)	Subcutaneously into the fracture site 0.4 mg/kg/day 7 days	Enhanced bone healing and improved microstructural parameters of the callus.	Zhao et al. [160]

## 6. Safety

Cardiovascular drugs, when administered systemically, can also impact wound healing processes depending on the duration of treatment. For instance, the study performed by Penington examined risk factors for infection and bleeding after skin lesion excision, involving 924 cases [162]. It identifies ulceration and antihypertensive use as significant risk factors for wound infection, with antihypertensive drugs increasing infection risk by 2.5 times. However, the study did not specify which antihypertensives caused this outcome. Another study highlighted the importance of distinguishing between the effects of various classes of antihypertensive drugs on wound healing, emphasizing the need for a nuanced understanding of their specific impacts [163].

Different classes of antihypertensive medications may impact wound healing outcomes variably. An in vitro study investigated five different antihypertensive drugs (metoprolol, amlodipine, ramipril, hydrochlorothiazide, and candesartan) using human skin cell models. It focused on their effects on cell metabolism and migration of fibroblasts and keratinocytes in 2D and 3D wound healing models [164]. The results showed that hydrochlorothiazide and ramipril inhibited cell processes, while candesartan and amlodipine had slight positive impacts. β-blockers like metoprolol had mixed effects. The therapeutic potential of antihypertensive calcium channel blockers, such as benidipine, in promoting wound healing has been explored, showing positive effects on wound closure in animal models [165].

Not only is the choice of cardiovascular drug crucial, but also the route of administration, dosage, and duration of treatment. As mentioned in the Introduction, untreated hypertension and hypercholesterolemia negatively impact wound healing. However, chronic use of cardiovascular drugs may also influence this process. An analysis of 9036 patients who underwent breast reconstruction after mastectomy between April 2015 and December 2018 divided into sartan, non-sartan, control, and non-hypertension groups [166]. Patients in the sartan group had the longest hospital stays, the highest rates of surgical complications, and the highest complication-related medical costs for both direct-to-implant and abdomen-based autologous reconstructions. Specifically, sartan use was found to be an independent risk factor for surgical complications in direct-to-implant reconstruction and for increased complication-related medical costs in abdomen-based autologous reconstruction. A meta-analysis performed by Liu et al. [163], on the other hand, showed the benefits of long-term antihypertensive systemic treatment on postoperative wound healing and scar formation in patients undergoing major non-cardiac surgery. The meta-analysis included patients treated with ACE inhibitors, beta-blockers, sartans, calcium channel blockers, diuretics, and multiple antihypertensive treatments across different types of major non-cardiac surgeries [167,168,169,170,171,172]. Specifically, the analysis showed that patients on long-term antihypertensive therapy had lower REEDA (Redness, Edema, Ecchymosis, Discharge, and Approximation) scores one-week post-surgery, indicating better initial wound healing, with reduced inflammation, better wound closure, and less redness and swelling. Additionally, these patients exhibited lower Manchester Scar Scale scores two months after surgery, suggesting improved cosmetic outcomes with less noticeable and better-formed scars. The study conducted from June 2015 to June 2018 examined whether chronic oral administration of ACEI and sartans influenced scar formation post-thyroid tumor surgery in 347 patients [173]. The results indicate that patients taking ACEI and sartans chronically may develop smaller scars compared to those using other antihypertensive drugs or no treatment, suggesting a potential therapeutic role in scar management.

Sometimes, medications used orally might cause side effects that are not caused after topical use. Gingival overgrowth is a well-documented side effect associated with the use of calcium-channel blockers [174]. This adverse effect has been reported in patients taking medications such as amlodipine, nifedipine, and verapamil [175]. The mechanism behind drug-induced gingival overgrowth involves the inhibition of calcium-ion influx across the cell membrane, affecting gingival fibroblasts [176]. Emampanahi et al. investigated the local effects of topical nifedipine, applied as a mucosal adhesive, on wound healing in the palate following gingival surgery [177]. In the triple-blind clinical trial enrolling 31 patients, 14 in the experimental group and 17 in the control group, wounds were standardized using mucotomies followed by free gingival grafting. Wound closure, healing criteria, and pain were assessed on days 2, 4, 7, 14, and 30 post-surgery. The results showed no significant differences in wound healing or pain reduction between the nifedipine-treated and control groups. While systemic side effects such as gingival overgrowth are known with calcium channel blockers, studying their local effects through a clinical trial is justified and necessary. It provides valuable insights into the drug’s potential benefits and risks in specific clinical contexts, such as wound healing after gingival surgery, which may differ from its systemic effects.

There are limited studies examining the systemic absorption of cardiovascular drugs administered topically. For instance, a study investigating 1% valsartan gel for wound healing in mice and diabetic pigs reported peak plasma concentrations of 50 nanomolar in porcine models [36]. However, these levels were transient, ranging from 1 to 50 nanomolar initially and becoming undetectable later during treatment. Compared to oral administration in humans, where valsartan levels typically range from 4000 to 5000 nanomolar, the lower and brief systemic exposure observed in this study suggests minimal impact on blood pressure regulation. In another prospective observational study [178], topical timolol 0.5% gel was administered to two distinct groups: patients with chronic wounds and those with glaucoma. At one hour post-application, plasma timolol concentrations were measured, revealing mean levels of 0.29 ng/mL in the chronic wound group and 0.43 ng/mL in the glaucoma group. These results indicate similar absorption rates between the groups, suggesting comparable bioavailability from topical application. Importantly, the observed plasma concentrations in both groups were below the threshold associated with systemic beta-blockade effects (10–40 ng/mL), indicating a low risk of systemic adverse effects, such as bradycardia or hypotension, with topical timolol use in chronic-wound and glaucoma patients. While these findings suggest safety in terms of systemic effects, further studies are warranted to explore topical cardiovascular drugs in various wound types and patient populations in the future.

There is a need for innovative solutions in wound healing treatment to overcome the challenges associated with various types of wounds, including chronic wounds. In the context of increasing bacterial resistance and the search for new solutions in wound treatment, drug repositioning has emerged as a promising strategy. Drug repositioning involves identifying new therapeutic uses for existing drugs, offering a cost-effective and time-efficient approach to drug discovery [179]. New polymers and structural scaffolds are also welcomed in wound treatment to enhance biocompatibility, improve mechanical properties, enable controlled drug release, and actively stimulate cellular activity for better healing outcomes [180,181]. These innovative approaches aim to create a conducive environment for wound healing, stimulate tissue regeneration, and prevent infections, ultimately leading to enhanced patient outcomes and reduced healing time.

## 7. Summary of Clinical Trials and Case Studies

Clinical trials have confirmed the effectiveness of statins, sartans, and beta-blockers in treating wounds. Timolol has been the subject of the most extensive research. Table 5 presents both clinical trial results and case studies, highlighting their complementary roles in understanding the therapeutic aspects of these medications in wound healing.

## 8. Conclusions

In conclusion, the contemporary approach to wound care extends beyond the exploration of novel pharmaceuticals, recognizing the potential of repurposing existing medicinal products such as beta-blockers, statins, and sartans. These drugs are traditionally used for other medical indications, and they emerge as valuable candidates in supporting wound healing processes. Their multifaceted actions, including anti-inflammatory and antimicrobial properties, make them promising contributors to wound management. In an era of antibiotic resistance, the antibacterial activity of these medicinal product classes assumes significance. The imperative for innovative solutions, such as electrospinning and nanoparticle technologies, becomes evident as we navigate the challenges of antibiotic-resistant infections. Research findings underscore the potential of beta-blockers, statins, and sartans, shedding light on their role in the evolving landscape of wound therapeutics. As the search for new developments in wound care continues, utilizing the various capabilities of existing pharmaceutical drugs offers a strategic way to advance treatment methods and meet the demands of the difficult topic of wound care.

## Figures and Tables

**Table 1:** Animal studies that testes the efficacy of sartans in wound healing.

Drug	Model	Method of Administration/ Dosage/ Duration of	Effect	Author
Losartan	Diabetic ale mice C57BL/6J, 8–10 weeks	Oral 10 mg/kg, daily 14 days	Accelerated wound repair, reduced inflammation, normalized tissue remodeling, improved wound healing, reduced fibrosis, enhanced extracellular matrix remodeling, increased vascularization	Kamber et al. [34]
Valsartan	AT1aR knock-out (AT1aR^−/−^) mice (based on C57BL/6J strain)	Study on Ang II type 1 receptor (AT1R) knock-out mice	Enhanced cell migration in vitro, improved wound closure in AT1R knock-out mice, promotion of keratinocyte and fibroblast migration, activation of EGFR signaling pathway, HB-EGF shedding	Yahata et al. [35]
Valsartan	Diabetic (36 months) Yucatan miniature swine	Topical 1/5/10% 5% valsartan gel, daily 57 days	Accelerated wound healing, suppression of inflammatory cytokines, upregulation of TGF-β1, promotion of collagen deposition, increased tensile strength, enhanced mitochondrial content, activation of molecular markers	Abadir et al. [36]
Losartan	Male Sprague-Dawley rats (8–12 weeks old, average weigh 225 g)	Oral losartan 50 mg/kg daily 14 days	Reduced fibroblast proliferation, suppressed collagen and TGF-β1 expression, downregulated phosphorylation of SMAD2/3 and TAK1 pathways, enhanced re-epithelialization and neovascularization	Fang et al. [37]
Losartan	C57BL/6 mice (average weight 25 g)	Topical 5% losartan cream daily 28 days	Inhibition of TGF-β1, collagen, and Smad expression; reduced Smad phosphorylation; exhibited superior anti-scarring effects	Zhao et al. [38]
Valsartan	New Zealand albino male rabbits	Oral administration 0.75 mg/kg/day 40 days	Inhibition of pathological scar formation, reduction in fibroblast count and epithelial thickness,	Kurt et al. [39]
Losartan	Rabbits weighing approximately 2.5–3.0 kg	Microneedle patches 0.05–50 mg/mL 15 days	inhibition of hypertrophic scar fibroblasts proliferation and migration, reduction in scar hyperplasia, decreased scar elevation index, inhibition of collagen deposition	Huang et al. [23]
Valsartan	Male, ten-week-old rats	Topical 1% valsartan filament hydrogel at day 10 and day 13 24 days	Accelerated wound closure, downregulated TGF-β signaling pathway mediators, increased mitochondrial metabolic pathway intermediates	Nidadavolu et al. [44]
Valsartan	Adult male Sprague-Dawley rats (180–200 g)	Topical 2% valsartan solid lipid nanoparticles integrated hydrogel gel twice a day 12 days	Sustained drug release, antibacterial effects, improved wound healing pathways (COX-2, NF-κB, NO, TGF-β, MMPs, VEGF)	El-Salamouni et al. [47]
Valsartan	Male Wistar rats 12 weeks (140–150 g)	Topical PLA/HA–valsartan hydrogel scaffold (1–1.5%) daily 14 days	Enhanced re-epithelization rate, reduced inflammatory cell infiltrates, better than conventional treatment and scaffolds with ascorbic acid	Ilomuanya et al. [46]

**Table 2:** Animal studies that tested the efficacy of beta-blockers in wound healing.

Drug	Model	Method of Administration/ Dosage/ Duration of Treatment	Findings	Author
Dexpanthenol and nebivolol	Albino Wistar rats (250–300 g)	Topical 5% nebivolol cream, daily 21 days	Both dexpanthenol and nebivolol groups showed higher wound healing rates than the control group, with no significant difference between them.	Ulger et al. [59]
Nebivolol	Albino Sprague-Dawley rats (150–200 g)	Topical Chitosomal suspension with nebivolol in the amount covering the wound 15 days	Significantly higher fibroblast proliferation compared to drug suspensions and the blank formula. In vivo evaluation showed superior efficacy in wound healing.	Elsherif et al. [60]
Propranolol	Diabetic mice wound model	Topical 1% propranolol cream, daily 21 days	Increased epidermal growth factor (EGF) protein expression, regulated angiogenesis, lower vascular endothelial growth factor (VEGF) expression, and increased NG2 proteoglycan.	Zheng et al. [61]
Timolol, propranolol	Wistar rat burns model	Oral (propranolol) and topical (timolol) 5.5 mg propranolol/1% timolol cream, daily 14 days	Propranolol did not show advantages in necrosis prevention and wound contraction and healing, and it did not reduce oxidative stress; it impaired keratinocyte migration, and promoted ulceration, chronic inflammation, and fibrosis, yet reducing the necrotic zone. Timolol effectively prevented necrosis, facilitated healing, and boosted antioxidant capacity.	Freiha et al. [62]
Propranolol	Mice wound model	Topical 0, 2% or 4% propranolol incorporated into electrospun nanofibrous wound dressing mats, daily 14 days	Significantly reduced wound size, improved histopathologic characteristics, and lowered oxidative stress after 14 days.	Zaeri et al. [71]
Nebivolol	Diabetic Sprague-Dawley rats wound model	Topical 5% nebivolol-loaded microsponge gel 10 days	Significant wound closure by day 10, supported by histological results. Extended-release drug microsponge gel provides an optimal moist wound-healing environment, demonstrating effective wound healing in diabetic rats.	Pandit et al. [90]

**Table 5:** Clinical trials and case studies that evaluated treatments in wound healing.

Drug	Type of Study	Study Group	Treatment	Findings	Author
Simvastatin	A double-blind, randomized, placebo-controlled clinical trial	76 patients	Oral/topical 20 mg/day of oral simvastatin/simvastatin 1% topical solution 8 weeks	Topical simvastatin was associated with a greater decrease in acne severity compared with oral and placebo groups.	Ahmadvand et al. [121]
Timolol	A multicentric Study	116 patients (58 patients with rosacea and 58 patients with acne)	Topical 0.5% drops with timolol maleate 8 weeks	Efficacy in the treatment of acne, especially in non-inflammatory lesions, more effective in erythematous-pustular rosacea than papulopustular rosacea, with negligible side effects.	Mokadem [182]
Timolol	A multicentric Study	39 patients	Topical 0.5% drops with timolol maleate, with dressing changes twice a day, once a day, every other day, or continuous application At least 4 weeks	34 wounds out of 55 healed completely, 15 wounds showed improvement in terms of wound reduction, 4 wounds remained unchanged, and 2 wounds worsened.	Cahn et al. [71]
Losartan	A pilot placebo-controlled single-blind study	30 adults	Topical 5% losartan ointment, twice daily 3 months	Alleviation of keloid and hypertrophic scars; and reduction in vascularity and pliability.	Hedayatyanfard et al. [40]
Timolol	A prospective study	100 patients	Topical Timolol 0.5% solution, at 1, 3, and 7 days 30 days	The topical application of timolol demonstrated a reduction in ulcer size on days 15 and 30.	Valluru et al. [77]
Losartan	A randomized clinical trial	46 patients	Topical 5% ethosomal gel, twice daily 12 weeks	Investigating the efficacy of treating keloids, comparison with triamcinolone acetonide injections.	Anggraini et al. [41]
Timolol	A randomized controlled trial	20 patients	Topical Timolol 0.5% solution, every 2 days 4 weeks	A mean reduction in ulcer size of 86.80% with timolol and 43.82% with saline after 4 weeks.	Rai et al. [81]
Atorvastatin	A randomized double-blind placebo-controlled clinical trial	60 patients	Topical 1% atorvastatin-loaded nanoemulgel, twice a day 14 days	Accelerated laparotomy surgical wound healing and relieved post-operative pain.	Saghafi et al. [128]
Simvastatin	A randomized double-blind placebo-controlled clinical trial	40 patients	Intra-oral topical application 10 mg/mL simvastatin/chitosan gel, three times/day 14 days	Reduced discomfort and improved wound healing in palatal donor site following free gingival graft procedures in individuals with periodontal conditions.	Madi et al. [114]
Timolol	Case report	2 patients who developed hypergranulation	Topical 0.5%timolol maleate ophthalmic gel forming solution, twice daily 14 days	Hypergranulation, unresponsive to standard care, resolved after treatment with topical timolol, and re-epithelialization of the surgical sites was observed.	Waldman et al. [74]
Timolol	Case report	43-year-old woman presented with a large refractory wound	Topical 0.5% timolol maleate solution, daily 8 weeks	Mean percentage change in ulcer area at 4, 8, and 12 weeks substantially higher compared to the control group.	Tang et al. [75]
Timolol	Case report	Two one-year-old children with chronic wounds in the nail bed and nuchal crease	Topical Timolol 0.5% solution, twice daily 8 weeks	Significant healing (100% and 80%) was achieved after 3 and 8 weeks in two cases of blistering epidermal separation.	Chiaverini et al. [76]
Propranolol	Case study	A 68-year-old patient with a deep ulcerating lesion	Topical 1% propranolol cream, three times/day 3 weeks	Significant improvement and complete healing within four weeks.	Vestita et al. [63]
Timolol	Case study	68-year-old woman undergoing confluent CO_2_ laser ablation with fractional laser	Topical 0.5% gel-forming solution 14 days	Rapid epithelialization and healing over the course of 8 weeks.	Joo et al. [72]
Timolol	Case study	40-year-old male	Topical Timolol 0.5% solution, 3 times/day 6 weeks	Reduced inflammation and pain and significant wound closure within six weeks.	Alsaad et al. [79]
Timolol	Case–control study	60 patients with chronic leg ulcers	Topical Timolol 0.5% solution, every 2 days 12 weeks	The mean percentage change in ulcer area at 4, 8, and 12 weeks was substantially higher in the group treated with topical timolol (25.29%, 43.77%, and 61.79%) compared to the control group (11.92%, 22.40%, and 29.62%).	Thomas et al. [78]
Timolol	Observational prospective Study	82 patients	Topical Timolol 0.5% solution, daily 6 weeks	Significant improvement in area reduction (up to 98.75%) was noted in all groups except diabetic and pressure ulcers. Patient satisfaction was high, and adverse effects were mild to moderate.	Vestita et al. [82]
Timolol	A phase-II randomized controlled study	43 patients	Topical Timolol maleate gel (Timoptol^®^ LP 0.5%), every 2 days 12 weeks	≥40% reduction in ulcer area by week 12. The results indicated that 67% of timolol-treated patients met this endpoint, compared to 32% in the control group. No serious adverse events were reported.	Baltazard [83].
Nifedipine	A randomized, double-blind, placebo-controlled clinical trial	200 critically ill patients with stage I or II pressure ulcers	Topical 3% ointment 14 days	Accelerated healing and a greater reduction in ulcer stage and surface area.	Zolfagharnezhad et al. [148]
Lisinopril	Case study	71-year-old male patient with a venous leg ulcer	Oral lisinopril treatment	Delayed wound healing.	Buscemi et al. [155]
Captopril	Case report	18-year-old female patient	Topical 5% solution in cold cream, twice daily for 6 weeks	Marked improvement in keloid: reduced lesion height, eliminated redness and scaling, reduced itchiness. No cutaneous or systemic side effects observed.	Ardekani et al. [157]
Enalapril	Case study	40 patients with multiple keloids w	Intralesional injection 0.125 mg/mL, 30 units/session 3 sessions monthly 3 months	Significant improvement in keloid appearance.	Iannello et al. [158]
